# The Antiproliferative Effect of Chloroform Fraction of *Eleutherine bulbosa* (Mill.) Urb. on 2D- and 3D-Human Lung Cancer Cells (A549) Model

**DOI:** 10.3390/ph16070936

**Published:** 2023-06-28

**Authors:** Nur Hannan Zakaria, Norazalina Saad, Che Azurahanim Che Abdullah, Norhaizan Mohd. Esa

**Affiliations:** 1 UPM-MAKNA Cancer Research Laboratory (CANRES), Institute of Bioscience, Universiti Putra Malaysia, 43400 Serdang, Selangor, Malaysia; nurhannan25296@gmail.com (N.H.Z.); norazalina@upm.edu.my (N.S.); azurahanim@upm.edu.my (C.A.C.A.); 2 Natural Medicine and Product Research Laboratory (NaturMeds), Institute of Bioscience, Universiti Putra Malaysia, 43400 Serdang, Selangor, Malaysia; 3 Department of Physics, Faculty of Science, Universiti Putra Malaysia, 43400 Serdang, Selangor, Malaysia; 4 Materials Synthesis and Characterization Laboratory (MSCL), Institute of Nanoscience and Nanotechnology (ION2), Universiti Putra Malaysia, 43400 Serdang, Selangor, Malaysia; 5 Department of Nutrition, Faculty of Medicine and Health Sciences, Universiti Putra Malaysia, 43400 Serdang, Selangor, Malaysia

**Keywords:** *Eleutherine bulbosa*, chloroform fraction, A549 cells, anticancer, antiproliferative

## Abstract

Since lung cancer is the leading cause of cancer-related death worldwide, research is being conducted to discover anticancer agents as its treatment. *Eleutherine bulbosa*, a Dayak folklore medicine, exhibited anticancer effects against several cancer cells; however, its anticancer potency against lung cancer cells has not been explored yet. This study aims to determine the anticancer potency of *E. bulbosa* bulbs against lung cancer cells (A549) using 2D and 3D culture models, as well as determine its active compounds using gas chromatography-mass spectrometry (GC-MS) analysis. Three fractions of *E. bulbosa* bulbs, namely chloroform, n-hexane, and ethyl acetate, were tested for cytotoxicity using 3-(4,5-dimethylthiazol-2-yl)-2,5-diphenyltetrazolium-bromide (MTT) and CellTiter-Glo. The antiproliferative effects of the most cytotoxic fraction against the 2D culture model were determined by a clonogenic survival assay and propidium iodide/Hoechst 33342 double staining, whereas the effects against the 3D culture model were determined by microscopy, flow cytometry, and gene expression analysis. The chloroform fraction is the most cytotoxic against A549 cells than other fractions, and it inhibited colony formation and induced apoptosis of A549 cells. The chloroform fraction also inhibited the growth of the A549 spheroid by suppressing the spheroid size, inducing apoptosis, reducing the proportion of CD44 lung cancer stem cells, causing arrest at the S phase of the cell cycle, and suppressing the expression of the *SOX2* and *MYC* genes. Furthermore, the GC-MS analysis detected 20 active compounds in the chloroform fraction, including the major compounds of eleutherine and isoeleutherine. In conclusion, the chloroform fraction of *E. bulbosa* bulbs exhibit its antiproliferative effect on 2D and 3D culture models of A549 cells, suggesting it could be a lung cancer chemopreventive agent.

## 1. Introduction

Lung cancer is the second most diagnosed cancer following breast cancer and the leading cause of cancer deaths globally [1]. In Malaysia, according to the National Strategic Plan for Cancer Control Programme 2016–2020, lung cancer is the third most diagnosed cancer and the leading cause of cancer-related mortality [2]. As the name depicts, lung cancer is a malignant tumour with uncontrolled cell growth that forms in the lung tissues and is mainly divided into two types which are small-cell lung (SCLC) and non-small-cell lung cancers (NSCLC) [3,4].

Depending on the types and stages of lung cancer, its treatment options include surgery, radiotherapy, chemotherapy, and immunotherapy [5]. As for chemotherapy, cisplatin is the most widely used drug for treating lung cancer. Still, severe adverse reactions and drug resistance remain the main obstacles to successful lung cancer treatment [6]. Thus, it is essential to identify and develop more effective lung cancer treatments with fewer disadvantages [7].

In folklore medicine, natural products have been used to treat many diseases, including lung cancer [8]. Hence, it is unsurprising that many pharmaceutical drugs are derived from natural products of various medicinal plants, including approximately 50% of chemotherapeutic agents [9]. Therefore, traditional herbal medicine (THM) is a promising approach for lung cancer therapy as it could reduce chemotherapy-related adverse effects, extend survival, and enhance the tumour response in NSCLC patients when used as an adjuvant therapy with conventional chemotherapy [10]. However, only a few medicinal plants were scientifically screened for their anticancer properties, including *Eleutherine bulbosa* [11].

*Eleutherine bulbosa* (Miller), known as the Urban or Dayak onion, is a herbaceous plant from the Iridaceae family. Although it is a native flora of Indonesia, it has been widely cultivated in Southern America, the African region, Southeast Asia, and other tropical countries [12,13,14]. Many other scientific names in the literature, including *Eleutherine americana* (Aubl.) Merr. ex K. Heyne and *Eleutherine plicata* Herb. ex Klatt. have been used to refer to the *Eleutherine bulbosa* species (Mill.) Urb. However, according to the databases of Kew and the Missouri Botanical Garden, *Eleutherine bulbosa* is the scientific name presently accepted for this species [15]. This plant is a seasonal perennial with a red bulbous rootstock, tiny, white, stellate flowers, and cauline, sheathing leaves. Its medicinal properties can be found in fleshy bulbs enclosed with reddish tunics [16]. The bulbs contain a high concentration of bioactive components, such as phenol, flavonoids, steroids, alkaloids, terpenoids, and saponins which can be obtained through extraction [17]. It has been shown that this plant exhibits its cytotoxic effect on several cancer cells, such as colon and ocular cancer cells, suggesting that it has potential as an anticancer drug [18,19]. 

However, the preclinical studies specifically evaluating the potential anticancer effect of *E. bulbosa* bulbs are still limited. Since this plant exhibits potent anticancer activity against ocular cancer cells, as reported previously by our group, it could also be effective as an alternative treatment for lung cancer [20]. Thus, this present study aims to evaluate the anticancer effects of *E. bulbosa* bulbs on lung cancer cells (A549) using 2D and 3D models and determine the presence of active compounds that may contribute to its anticancer effects. 

The cytotoxic effects of *E. bulbosa* bulbs on lung cancer cells were initially screened using a 2D culture model; however, due to the inability to mimic the in vivo environment, the 3D cell culture model was used for a secondary screening due to its better capacity to simulate the in vivo environment and produce more accurate data on the cytotoxic effects of *E. bulbosa* on lung cancer cells [21,22]. This study focused on the bulbs rather than other parts of the plant because the bulbs, as reported in previous studies, contain a higher concentration of the bioactive components with strong cytotoxic potential against various cancer cells [17,23]. This study is the first to present the result of the antiproliferative effects of *E. bulbosa* bulbs on lung cancer cells using 2D and 3D culture models. The novel findings of this study contribute to a deeper understanding of the potential of *E. bulbosa* bulbs as a promising treatment option for lung cancer.

## 2. Results and Discussion

### 2.1. 2D Culture Condition

#### 2.1.1. Cytotoxic Activities of *E. bulbosa* Fractions and Cisplatin on A549 and MRC-5 Cells

The cytotoxic effects of chloroform, n-hexane, and ethyl acetate fractions of *E. bulbosa* bulbs were tested against A549 cells using the MTT assay. This colorimetric-based assay reduces the MTT tetrazolium salt to an MTT formazan product by the succinate dehydrogenase enzyme after being taken up by the mitochondria of living cells [24].

**Figure 1:**
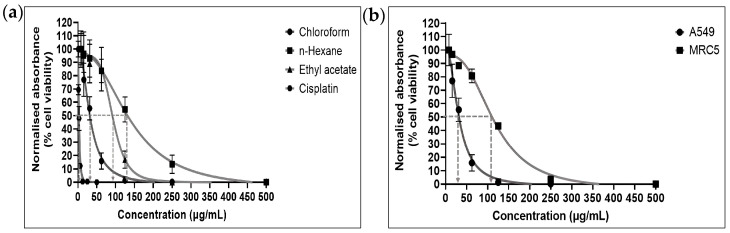
Cytotoxic effects of different fractions of *E. bulbosa* and cisplatin against A549 and MRC-5 cells after 72 h of treatment. (**a**) Viability of A549 cells against increasing concentrations of three different fractions of *E. bulbosa* (n-hexane, chloroform, and ethyl acetate) and cisplatin. (**b**) Cytotoxicity of the chloroform fraction on A549 and MRC-5 cells in concentrations ranging between 0–500 ug/mL. The data were recorded as the mean ± standard deviation (SD) from three independent experiments.

Figure 1a shows the cytotoxic effects of each fraction of *E. bulbosa* against A549 cells after 72 h of treatment, in which the chloroform fraction was the most cytotoxic fraction, followed by ethyl acetate and n-hexane fractions on A549 cells. The relationship between the IC_50_ value and the cytotoxic activity of a compound was inversely proportional, indicating that a lower IC_50_ value represents a higher cytotoxic activity of the extract [25]. In drug therapy, a low IC_50_ value is favoured as it indicates that the treatment is effective while exhibiting minimal systemic toxicity when given to the patient [26]. Indeed, the IC_50_ values of n-hexane, chloroform, and ethyl acetate fractions were 126.60 ± 4.15 μg/mL, 30.01 ± 2.14 μg/mL, and 83.44 ± 1.31 μg/mL, respectively (Table 1). 

Generally, a compound is considered active when the IC_50_ value is less than 20 μg/mL, moderately and weakly active with IC_50_ values ranging between 20–100 and 100–1000 μg/mL, respectively, and inactive if the IC_50_ value is higher than 1000 μg/mL [25,27]. Therefore, in this study, chloroform and ethyl acetate fractions of *E. bulbosa* were moderately active against A549 cells with IC_50_ values of 30.01 ± 2.14 μg/mL and 83.44 ± 1.31 μg/mL, respectively, in the range of 20–100 μg/mL, whereas the n-hexane fraction was weakly active with IC_50_ values of 126.60 ± 4.15 μg/mL, in the range of 100–1000 μg/mL. The chloroform fraction exhibits the highest significant cytotoxic activity against A549 cells, with the lowest IC_50_ among the three *E. bulbosa* fractions (*p* < 0.0001).

Even though the chloroform fraction of *E. bulbosa* was the most cytotoxic against A549 lung cancer cells, it is vital to ensure that the fraction has minimal or no cytotoxic activity against normal cells. It is because, even though many current chemotherapeutic drugs can kill cancer cells, selective toxicity and severe side effects remain as unavoidable obstacles [28]. Hence, to determine whether the *E. bulbosa* chloroform fraction selectively targets cancer cells, its cytotoxic effect was also assessed on the normal lung cell line, MRC-5.

As shown in Figure 1b, the chloroform fraction of *E. bulbosa* reduced the viability of MRC-5 cells at a lower rate than the A549 cells. Furthermore, the chloroform fraction was moderately active against A549 cells with IC_50_ values of 30.01 ± 2.14 μg/mL but weakly active against the normal lung cells (MRC-5) with IC_50_ values of 102.2 ± 1.78 μg/mL (Table 2). The significant difference between IC_50_ values of the chloroform fraction against A549 and MRC-5 cells demonstrated that the chloroform fraction of *E. bulbosa* selectively killed cancer cells (*p* < 0.001). It is also supported by the calculated selectivity index (SI) of the chloroform fraction, which was 3.4 (Table 1). The tested compound is considered “selective” when the SI value is greater than 3 [29]. This index is widely used to indicate the ability of a tested fraction or compound to inhibit cancer cell development without damaging normal cells. Thus, in this study, the chloroform fraction of *E. bulbosa* showed selective toxicity on A549 cells (SI > 3) without affecting the MRC-5 cells. 

To determine whether the chloroform fraction of *E. bulbosa* was as cytotoxic as the commercial chemotherapeutic drug in current cancer therapy, the effect of cisplatin on cell viability of A549 cells was determined since cisplatin is the most common antineoplastic agent [30]. According to the United States National Cancer Institute Plant Screening Program, a pure compound was considered as cytotoxically active if its IC_50_ value was less than 4 μg/mL [31] Therefore, in our study, cisplatin was considered cytotoxically active against A549 cells after 72 h of treatment with IC_50_ values of 2.61 ± 0.09 μg/mL (Figure 1a). Despite its excellent capability in killing cancer cells, cisplatin is used restrictedly since it might have adverse effects on normal tissues [32]. A few studies have reported that cisplatin was not selective as it exhibited a high cytotoxic effect on the normal lung fibroblast cell line (MRC-5) [33,34]. Despite the lower cytotoxicity of the chloroform fraction of *E. bulbosa* compared to cisplatin, the chloroform fraction has a promising value as an anticancer agent as it is more selective in targeting lung cancer cells. Therefore, the effects of the *E. bulbosa* chloroform fraction on the survival and death of A549 cells were further evaluated using the IC_50_ value of 30 μg/mL as the tested concentration. 

#### 2.1.2. Effect of *E. bulbosa* Chloroform Fraction on Clonogenic Potential and Survival of A549 Lung Cancer Cells

Cancer cells can survive and proliferate as they are capable of forming colonies. These cells, which are referred to as clonogenic, are a subset of tumour cells within a tumour mass that can undergo self-renewal. As a result, it is impossible to fully eradicate these tumour-regrowing cells, making it one of the most frequent cancer treatment problems [35]. In this study, the clonogenic assay was performed to determine the anticancer effect of *E. bulbosa* chloroform fraction on A549 lung cancer cells after 10 days of treatment. This in vitro assay has been extensively used to evaluate the survival of cells, their ability to proliferate, as well as their induction of cell death [36,37]. It is a method commonly used to determine the efficacy of cytotoxic drugs over a long-term period [38]. 

**Figure 2:**
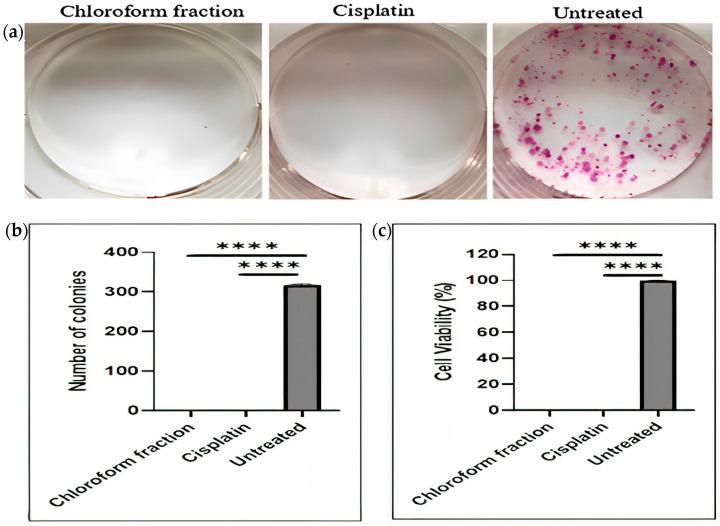
Effects of the chloroform fraction of *E. bulbosa* (30 ug/mL) and cisplatin (2.61 ug/mL) on A549 cells after 10 days of treatment. (**a**) Images of colony formations of A549 cells following treatment of cells with the chloroform fraction and cisplatin, (**b**) the number of A549 cell colonies and (**c**) cell viability after treatment with the chloroform fraction of *E. bulbosa* and cisplatin. The images in (**a**) are representative of two independent experiments (*n* = 2). The significant difference between groups was compared using an ANOVA. The level of significance is indicated as **** *p* < 0.0001. The untreated cells served as the positive control, and the results are represented as the mean ± SD from two independent experiments (*n* = 2).

Figure 2 shows the inhibitory effects of the *E. bulbosa* chloroform fraction and cisplatin on the proliferation and colony formation of A549 cells after treatment of 72 h. As shown in Figure 2a, the chloroform fraction of *E. bulbosa* completely inhibited the formation of A549 cells colonies after 10 days of treatment. Moreover, as shown in Figure 2b,c the chloroform fraction of *E. bulbosa* significantly inhibited the colony-forming ability and cell viability of A549 cells compared to the untreated control (*p* < 0.0001). Remarkably, at 30 μg/mL, the chloroform fraction of *E. bulbosa* was able to inhibit colony formation and viability of A549 cells.

#### 2.1.3. Effect of the *E. bulbosa* Chloroform Fraction on the Induction of Cell Death in A549 Cells 

Double staining of Hoechst 33342 and propidium iodide (PI) is a simple and common approach for determining the mode of cell death. Hoechst 33342 is a cell-permeable DNA-binding dye capable of staining the condensed chromatin of apoptotic cells more brightly than in normal cells. In contrast, PI is a cell impairment DNA-binding dye and can only pass through dead necrotic cells due to the plasma membrane rupture [39]. Therefore, the staining patterns that emerge from using these dyes simultaneously allow differentiation of normal, apoptotic, and necrotic cell populations [40]. In specific, cell nuclei that emitted only the blue Hoechst 33342 signal were deemed as live cells, whereas cell nuclei that emitted only the red fluorescent signal were categorized as necrotic cells [41]. On the other hand, cells that emitted both blue and red fluorescent signals are considered dead apoptotic cells, whereas live apoptotic cells were stained intensely blue [42].

**Figure 3:**
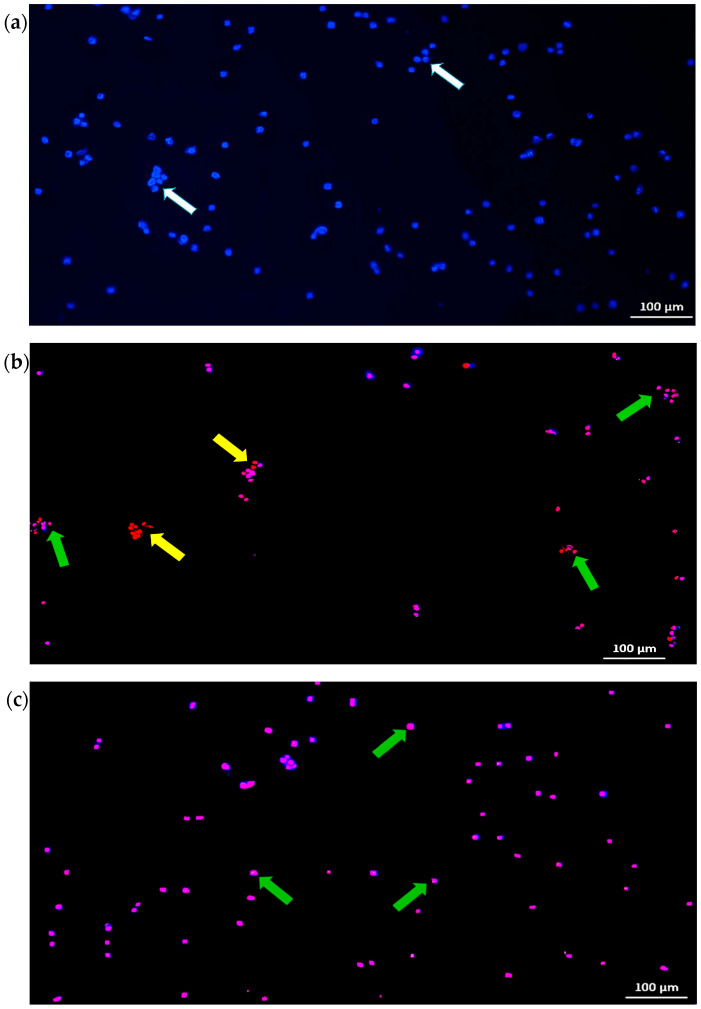
Induction of cell death following treatment of *E. bulbosa* with the chloroform fraction in A549 cells with Hoechst-33342/Propidium iodide double staining. (**a**) Fluorescence microscopic images of untreated A549 cell line; (**b**) Fluorescence microscopic images of A549 cells after 72-h treatment of cisplatin (2.61 μg/mL); (**c**) Fluorescence microscopic images of A549 cells after treatment of the chloroform fraction of *E. bulbosa* (30 μg/mL) for 72 h. Fluorescence images were captured at 5× magnification. White arrows show live cells (blue nucleus), green arrows show late apoptotic cells (pink nucleus), and yellow arrows shows necrotic cells (red nucleus). The scale bar represents 100 μm in length.

In this study, induction of apoptosis by the chloroform fraction of *E. bulbosa* (30 μg/mL) was determined in A549 cells after being treated for 72 h, with cisplatin serving as the positive control (2.61 μg/mL). The nuclei of all cells in the untreated group were stained blue, showing that all the cells were alive with normal and intact nuclei (Figure 3a). However, as shown in Figure 3b,c notable morphological changes were observed in the cell nuclei of A549 cells after treatment with the chloroform fraction of *E. bulbosa* and cisplatin compared to the untreated control. 

Following the cisplatin treatment of 2.61 μg/mL, the nuclei of A549 cells were stained pink and red, which indicate the presence of late apoptotic and necrotic cells, respectively (Figure 3b). In contrast, the A549 cells after treatment with the chloroform fraction of *E. bulbosa* were mainly dominated by a population of late apoptotic cells, as most cell nuclei were stained in pink (Figure 3c).

Although both apoptotic and necrotic deaths are parameters for the future study of potential drug candidates, apoptosis was more promising as it is a controlled and regulated cell death compared to necrosis [43]. In particular, apoptosis destroys undesirable cells without damaging the microenvironment, while necrosis produces severe inflammation of surrounding tissues due to spilling cell fluids into the peri-cellular space [44]. It is known that cancer therapies with anticancer agents and radiation might cause necrosis as an adverse effect of cancer treatment, as observed in cells treated with cisplatin in our study. Since necrosis in the tumour microenvironment is also linked to resistance against anticancer agents and radiation, it has become an essential clinical issue in cancer treatment [45]. Hence, the development of effective anticancer drugs and treatments should promote apoptosis without stimulating necrosis [46]. 

Therefore, as the chloroform fraction of *E. bulbosa* induced apoptosis of A549 cells, it may be promising as a candidate for the development of an anticancer agent.

### 2.2. GC-MS Analysis

**Figure 4:**
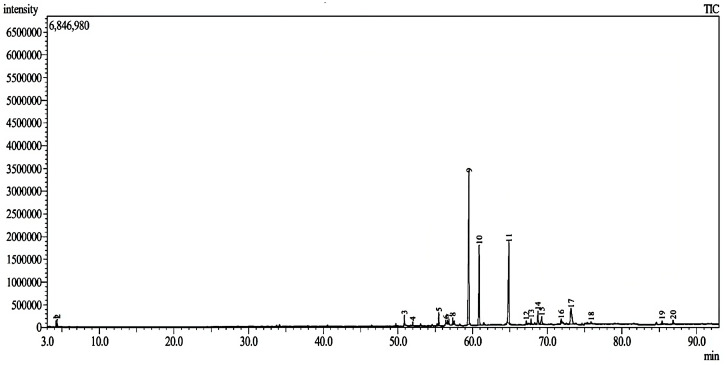
GC-MS chromatogram of the chloroform fraction of *E. bulbosa*. The GC-MS detected 20 peaks (1–20) in the chloroform fraction of *E. bulbosa*.

Overall, the chloroform fraction of *E. bulbosa* was found to be selectively cytotoxic to A549 lung cancer cells with observed inhibitory effects on colony-forming capability and cell proliferation whilst inducing apoptosis. To determine the chemical components that may contribute to promoting these anticancer activities, an GC-MS analysis was performed on the fraction. Previously, among the identified compounds of the chloroform fraction of *E. bulbosa* were hexadecanoic acid, 9,12-octadecadienoic acid, linolenic acid, octadecanoic acid, androstan-17-one, and 1-(2,3,5,6-tetramethyl-phenyl)-ethanone [47]. In this study, based on the GC-MS chromatogram of the chloroform fraction, 20 peaks were detected (Figure 4) and identified based on their retention time (RT) and similarity index (%) as listed in Table 2. 

Initially, the identification of components detected in the chloroform fraction of *E. bulbosa* was determined based on a reference library using the retention index (RI) and similarity index (SI). The spectral result is often set to have a threshold similarity index of ≥60 or 70% [48]. However, the GC-MS analysis only identified 12 compounds in the chloroform fraction of *E. bulbosa* with the SI ranging between 78% to 97%, while the remaining were not identified as their similarity index were less than 60%. In detail, eight observed peaks, which are peak numbers 5, 9, 10, 13, 14, 15, 17, and 18, could not be identified following mass spectra comparison with the mass spectral library (Table 2). Out of 12 identified components, it is presumed that hexadecanoic acid and 9,12-octadecadienoic acid may contribute to the cytotoxic effect of the chloroform fraction of *E. bulbosa* against A549 cells, as such findings were comparable to the previous study reported by Lestari et al. (2019) [47]. However, this hypothesis can only be tested by validating the anticancer effects of both compounds against A549 cells. 

**Figure 5:**
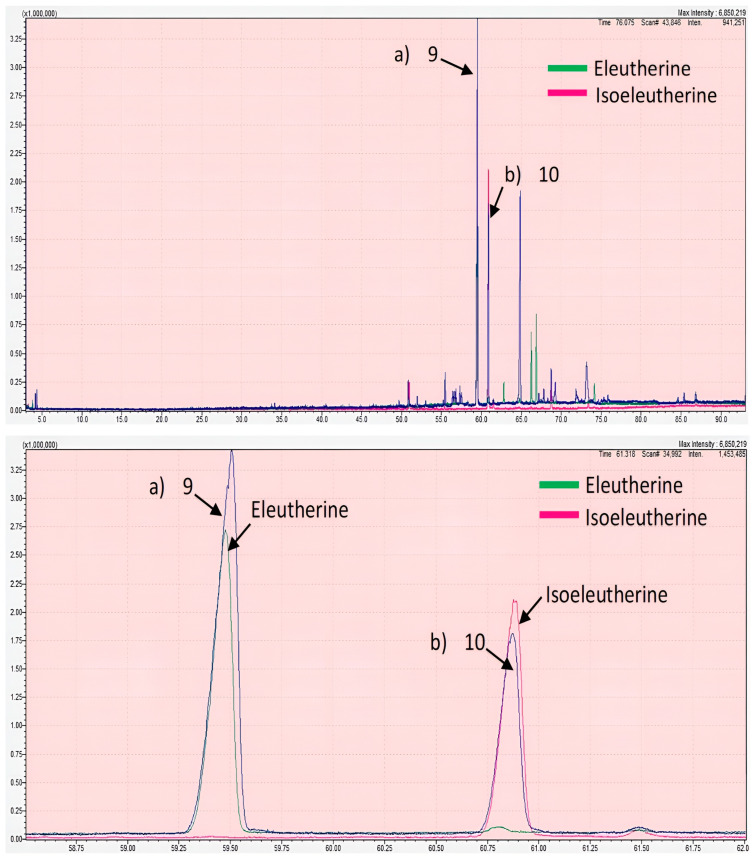
GC-MS chromatogram of chloroform fraction of *E. bulbosa* (**top**), eleutherine, and isoeleutherine (**bottom**). The two main peaks were detected at the same retention time of the standard compounds, which are (**a**) eleutherine and (**b**) isoeleutherine.

Besides the two identified compounds, it is possible that components detected as Peak 9 and Peak 10 in the chloroform fraction may also contribute to the observed anticancer effects on A549 cells. This is because Peak 9 and Peak 10 corresponded to major components of the fraction, with their percentages of the total peak area as 36.69% and 17.25%, respectively. Therefore, peaks 9 and 10 were further analyzed for identification by comparing the chromatographic peak retention indices with the standard compounds, which are eleutherine and isoeleutherine, under similar experimental conditions, as illustrated in Figure 5. The retention times of the unknown compounds at peaks 9 and 10 were 59.5095 and 60.8740 min, respectively, whereas those of standard compounds of eleutherine and isoeleutherine were 59.4793 and 60.8885 min, respectively. Except for the extremely small differences between the unknown peaks and standards, their retention times were nearly identical, which strongly suggested that Peak 9 and Peak 10 corresponded to eleutherine and isoeleutherine, respectively. This finding is consistent with a study published by Kamarudin et al. (2020), in which eleutherine was shown to be one of the bioactive compounds extracted from *E. bulbosa* [49]. However, to our knowledge, there were no reported biological activities of these two compounds.

Aside from previously mentioned chemical compounds, several studies reported that the chloroform fraction of *E. bulbosa* bulbs contained secondary metabolites such as alkaloids, flavonoids, quinone (naphthoquinone and anthraquinone), and azulenes [47,50,51]. These secondary metabolites have shown promising effects for treating cancer, especially lung cancer [52,53,54,55].

### 2.3. 3D Culture Condition

#### 2.3.1. Cytotoxic Activity of the Chloroform Fraction of *E. bulbosa* on Lung Cancer Spheroids

**Figure 6:**
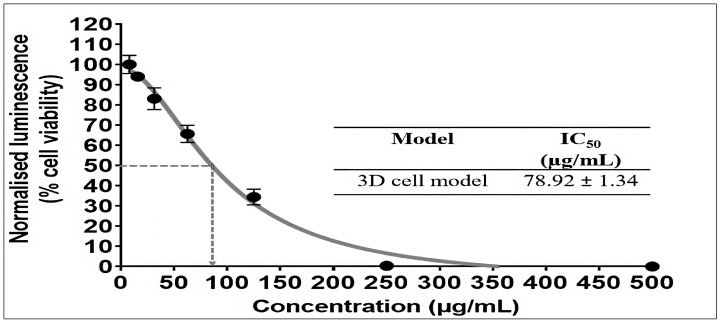
Cytotoxic effects of the chloroform fraction of *E. bulbosa* against the 3D cell model of A549 spheroids after 72 h of treatment. Results were represented as IC_50_ values (mean ± SD) of three independent experiments (*n* = 3).

The ability of the 3D culture model to mimic the primary features of an in vivo solid tumour has enhanced their usage for drug screening, and they may provide crucial information for predicting in vivo effectiveness [56,57]. The 3D culture model was used in this study as a secondary screening for determining the cytotoxic effect of the chloroform fraction of *E. bulbosa*. The CellTiter-Glo assay was used to assess the cytotoxic effect of the chloroform fraction of *E. bulbosa* on A549 cell 3D spheroids. The 72-h timeframe was used to standardize the treatment duration in both 2D and 3D culture models, ensuring that the experimental conditions were consistent and appropriate for assessing the cytotoxic effects of the chloroform fraction of *E. bulbosa* against the lung cancer cells. Figure 6 shows the cytotoxic effects of the chloroform fraction of *E. bulbosa* against the 3D culture model of A549 spheroids after 72 h of treatment, with the IC_50_ value being 78.92 ± 1.34 μg/mL. This finding showed that A549 cells cultured using a 3D culture model were less affected by the chloroform fraction of *E. bulbosa*, which may be related to the variable exposure between the cells on the exterior and interior [58]. Therefore, the cytotoxic effect of the chloroform fraction of *E. bulbosa* was better assessed using the 3D cell culture model due to its capacity to represent in vivo cellular conditions more accurately.

#### 2.3.2. Microscopy Analysis of Lung Cancer Spheroids

##### Effect of the Chloroform Fraction of *E. bulbosa* on the Size of the A549 Spheroid

**Figure 7:**
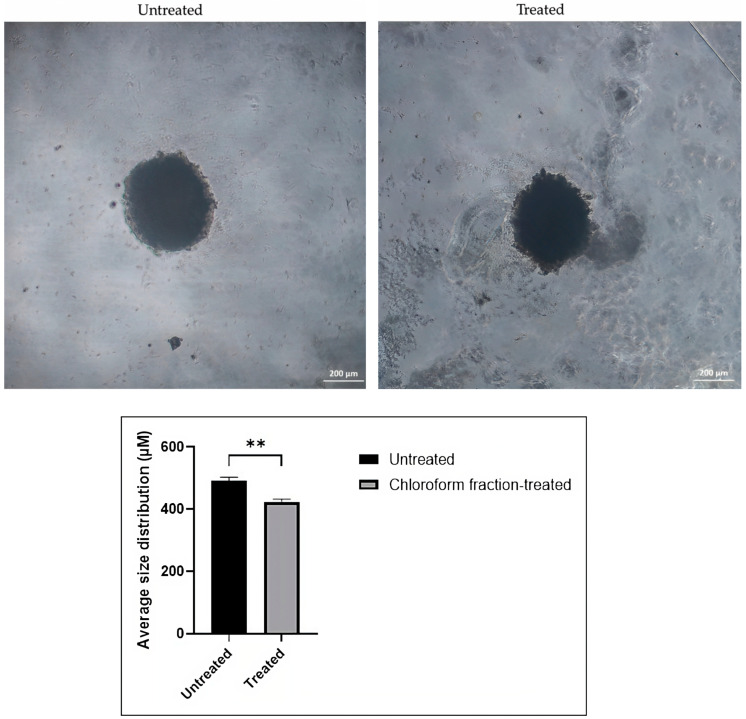
The effect of the chloroform fraction of *E. bulbosa* (78.92 μg/mL) on the size of A549 spheroids after 72 h of treatment. The chloroform fraction of *E. bulbosa* inhibited the growth of A549 spheroids by significantly reducing their size. The spheroids were captured using an inverted microscope (5× magnification), and the size of the spheroids was measured with ImageJ software. The scale bar represents 200 μm in length. The spheroid size was compared between untreated and chloroform fraction-treated spheroids using the Student’s *t*-test. The level of significance is indicated as ** *p* < 0.01. The data are presented as mean ± standard deviation (SD) (*n* = 3).

Spheroid size and shape measurements allow direct drug cytotoxicity monitoring over time [59]. The morphological changes of spheroids were observed under an inverted microscope, and the spheroids’ size differences between the untreated group and treatment group with the chloroform fraction of *E. bulbosa* were measured using ImageJ software. Figure 7 demonstrates that the A549 spheroids retained their round shape after treatment with the chloroform fraction of *E. bulbosa* at a concentration of 78.92 μg/mL. Cell binding or cross-linking with the extracellular matrix could be responsible for this maintenance of structure [60]. Spheroids should retain their uniformly round shape following treatment and thus be considered for morphological studies of the effects of anticancer drugs [61]. The size-based analysis can determine the outcome of drug testing treatments if spheroids have a round shape with a defined boundary [62]. The average size of spheroids after being treated with chloroform fraction of *E. bulbosa* was 422.58 ± 9.43 μm, whereas the average size of untreated spheroids was 492.36 ± 10.62 μm, summarized in Table 3. This finding showed that the size of the spheroids after treatment with the chloroform fraction of *E. bulbosa* was reduced significantly compared to the untreated group (*p* < 0.01), possibly due to their cytotoxic effects on the A549 spheroids. Thus, treatment with the chloroform fraction of *E. bulbosa* can inhibit the growth of A549 cells spheroids by reducing the size of the spheroids.

##### Cell Death Analysis of Spheroid Using Hoechst 33342/PI Staining

Hoechst 33342 and PI double staining were used to determine the mode of cell death, apoptosis, or necrosis in the A549 spheroid induced by exposure to the chloroform fraction of *E. bulbosa*.

**Figure 8:**
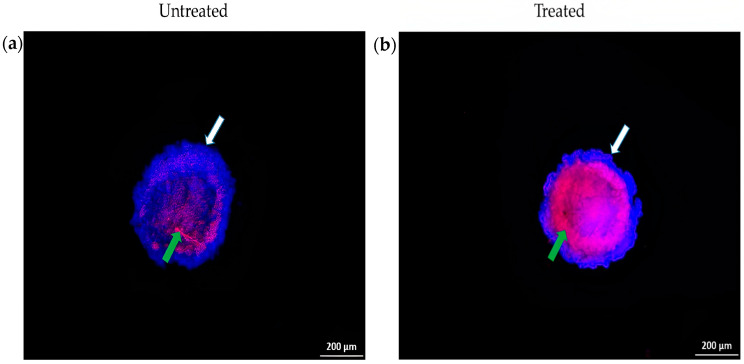
Induction of cell death by chloroform fraction of *E. bulbosa* in A549 spheroids using Hoechst-33342/Propidium iodide double staining. (**a**) Fluorescence microscopic images of the untreated A549 spheroid; (**b**) Fluorescence microscopic images of A549 cells after treatment of the chloroform fraction of *E. bulbosa* (78.92 μg/mL) for 72 h. Fluorescence images were captured at 5× magnification. White arrows show live cells (blue nucleus), and green arrows show late apoptotic cells (pink nucleus). The scale bar represents 200 μm in length.

Figure 8 shows the morphology of A549 spheroids examined under a fluorescence microscope after being stained with Hoechst 33342 and PI. The untreated cells served as a control group for this study. In control cells (shown in Figure 8a), the blue fluorescence is distributed uniformly throughout the nucleus, indicating that the cells are alive. Within the interior of the spheroid, a smaller population of cells has been stained pink, suggesting that these cells have undergone late apoptosis. This may have been caused by a reduced nutritional and metabolic diffusion from the spheroid’s exterior to its interior. As a result, the core of the spheroid became nutritionally deficient, eventually leading to the cells’ death [63]. Additionally, the core of the spheroid does not receive enough oxygen as the outside cells do, causing them to undergo hypoxia [64]. Hypoxia can trigger apoptosis by increasing the expression of the hypoxia-inducible factor (HIF), increasing concentrations of oxygen free radicals, and causing mitochondrial damage [65]. This finding showed that the presence of apoptotic cell death in the interior of the A549 spheroid demonstrated the occurrence of hypoxia. 

Figure 8b shows that spheroids treated with the chloroform fraction of *E. bulbosa* exhibited a small population of blue-stained cells in the exterior of the spheroid and a large population of pink-stained cells in the interior of the spheroid compared to the control group, due to the loss of cellularity in the spheroids. The results indicated that the exterior layer comprised viable cells, whereas late apoptotic cells dominated inside the spheroid core. Drug effectiveness depends on the compound’s capacity to bind and function at the outer layer of the spheroids, penetrate the interior layer, and enter tumour cells [66]. Therefore, the chloroform fraction of *E. bulbosa* has the potential to induce apoptosis in the A549 spheroid.

#### 2.3.3. Flow Cytometry Analysis on Lung Cancer Spheroids

##### Immunophenotyping of CD44 Lung Cancer Stem Cells

CD44 is a cell surface protein that plays an essential role in the function of cancer stem cells (CSCs) and in promoting the progression of various tumors [67]. CD44 expression may be used as an indicator of tumor subtype and a marker of cancer stem cells [68]. Tumorigenic transformations are triggered due to abnormal CD44 expression resulting from gene alteration [69]. 

**Figure 9:**
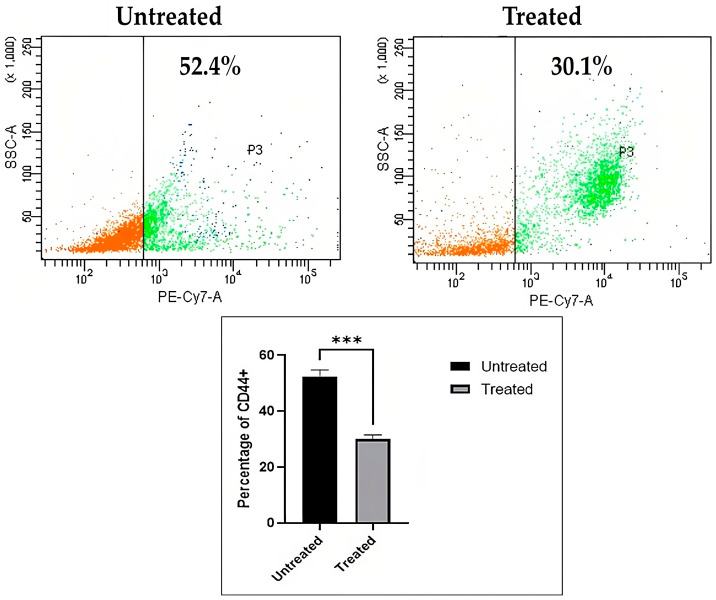
Immunophenotyping of CD44 markers on A549 spheroids. The percentage of CD44 after being treated with the chloroform fraction of *E. bulbosa* was lower than the untreated group in A549 spheroids. The mean difference between the two groups was determined using the Student’s *t*-test. The level of significance is indicated as *** *p* < 0.001.

To study the capacity of the chloroform fraction of *E. bulbosa* on the CD44 subpopulation of cells present inside the A549 spheroids to target and inhibit CSCs specifically, the expression of CD44 in A549 spheroids was analyzed using flow cytometry. Figure 9 demonstrates the percentage of cells that exhibited the CD44 marker in A549 spheroids. The percentage of CD44 was reduced significantly (*p* < 0.001) in the chloroform fraction of *E. bulbosa* treated group (30.1%) as compared to the untreated group (52.4%). This finding showed that the chloroform fraction of *E. bulbosa* was effective in targeting and decreasing CD44 expression in A549 spheroids. A reduction in CD44 expression may prevent the proliferation of A549 spheroids while simultaneously causing an increase in apoptosis. Hence, it suggests that the chloroform fraction of *E. bulbosa* might be a potential therapy for patients with CD44-expressing lung cancer. 

##### Cell Cycle Analysis

**Figure 10:**
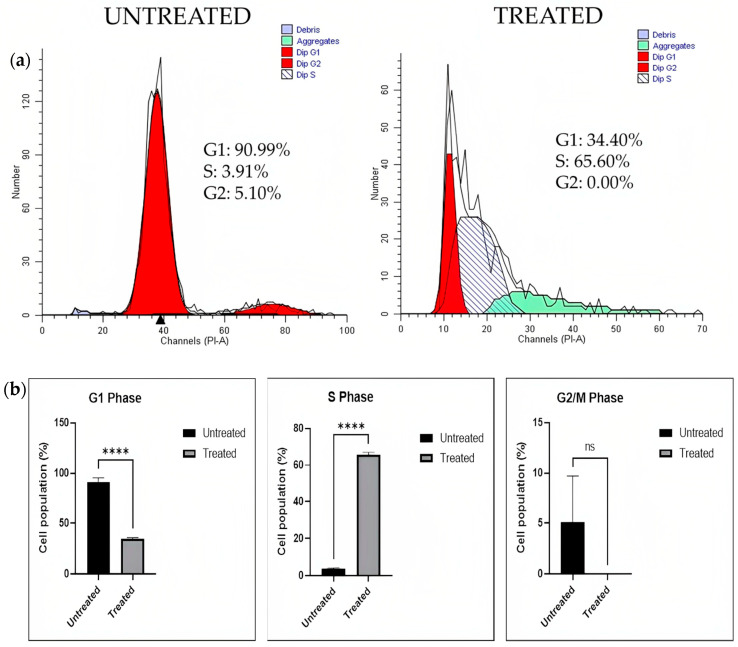
The distribution of A549 spheroid cells treated with the chloroform fraction of *E. bulbosa*. (**a**) Representative results of cell cycle analysis for untreated and treated A549 spheroid cells, (**b**) Statistical analysis of the percentage of A549 spheroid cells in the G1, S, and G2/M phases of the cell cycle. The mean difference between the two groups was determined using the Student’s *t*-test. The level of significance is indicated as **** *p* < 0.0001. All measurements were conducted in triplicate, and the data were expressed as the mean ± standard deviation.

The cell cycle profiles of the A549 spheroid after treatment with the chloroform fraction of *E. bulbosa* (78.92 μg/mL) were analyzed by flow cytometry after 72 h of exposure. Cell cycle distribution was analyzed to determine whether the chloroform fraction of *E. bulbosa* suppresses cell growth by disturbing the cell cycle. The untreated cells were utilized as a control. Figure 10 shows representative flow cytometry plots of untreated A549 spheroids and A549 spheroids treated with the chloroform fraction of *E. bulbosa*. The graphical representations used clustered bars to depict the G1, S, and G2/M values. 

This finding showed that treatment of the chloroform fraction of *E. bulbosa* in A549 spheroids significantly decreased the percentage of cells in the G1 phase (*p* < 0.0001) and significantly increased the percentage of cells in the S phase compared to the control group (*p* < 0.0001). It has been shown that the chloroform fraction of *E. bulbosa* caused cell arrest in the S phase and prevented cells from entering the mitotic phase. The S phase checkpoint is responsible for monitoring the progression of the cell cycle and inhibiting DNA synthesis when damaged DNA occurs [70]. However, the percentage of cells in the G2/M phase decreased non-significantly in the A549 spheroid after being treated with the chloroform fraction of *E. bulbosa* compared to the control group (*p* > 0.05), which indicated a lower level of cell division in response to the treatment. These findings showed that the chloroform fraction of *E. bulbosa* disrupts the mitotic phase by interfering with cell cycle checkpoints in response to DNA damage or is incompletely replicated. Therefore, the chloroform fraction of *E. bulbosa* has the potential to inhibit cell growth, arrest the cells at the S phase, and trigger apoptosis in the A549 spheroid.

#### 2.3.4. Inhibition of Gene Expression by the Chloroform Fraction of *E. bulbosa* in A549 Spheroids

The stem cell pluripotency-associated transcription factors, including *SOX2* and *c-MYC*, were initially found in the induced pluripotent stem cells and exhibited differential upregulated expression in A549 cell spheroids [71]. The upregulation expression of these pluripotent transcription factors may facilitate self-renewal and differentiation of stem cells [72]. The *c-MYC* gene is responsible for regulating proliferation, which is under the control of the *SOX2* gene [73]. The *c-MYC* promotes the transcriptional activity of the *SOX2* promoter by binding to the promoter of *SOX2*. Consequently, an upregulation in the expression of *SOX2* leads to an improvement in cancer stemness [74]. 

**Figure 11:**
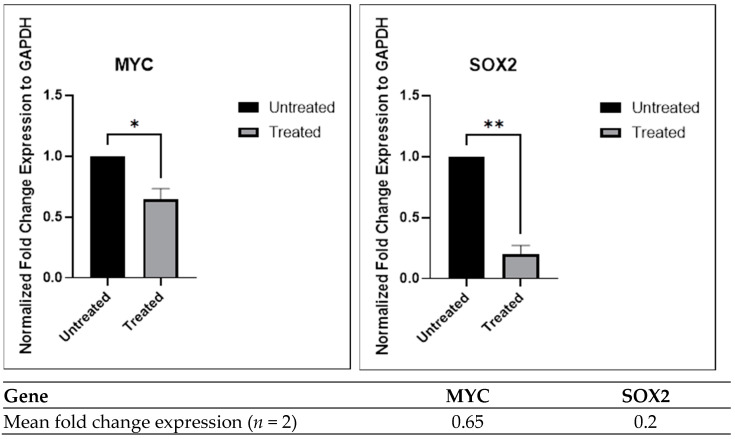
Normalized fold ratio of A549 spheroids. The relative expression of both pluripotency genes was quantified and normalized to the GAPDH level, and the expression levels were calculated using the 2^−ΔΔCt^ method. The mean difference between the two groups was determined using the Student’s *t*-test. The level of significance is indicated as * *p* < 0.05 and ** *p* < 0.01. The values are presented as the mean ± standard deviation from the two independent experiments (*n* = 2).

Evaluation of gene expression regulation in A549 spheroids, with a focus on the pluripotency genes *MYC* and *SOX2*, was performed using quantitative real-time PCR (RT-qPCR) to determine the effects of the chloroform fraction of *E. bulbosa*. The relative gene expression of the pluripotency genes in A549 spheroids after treatment with the chloroform fraction of *E. bulbosa* is shown in Figure 11. This finding showed that the expression of both pluripotency genes was significantly downregulated in A549 spheroids as determined by RT-qPCR analysis (*p* < 0.01). The ability of the chloroform fraction of *E. bulbosa* to downregulate the expression of *SOX2* and *MYC* genes by 0.2- and 0.65-fold, respectively in A549 spheroids suggests that it may be capable of targeting the CSCs subpopulation within tumor spheroids. These results suggest that the chloroform fraction of *E. bulbosa* can inhibit the growth of A549 spheroids by targeting and downregulating the expression of *SOX2* and *MYC* genes. 

## 3. Materials and Methods

### 3.1. Chemicals and Reagents

Chloroform, n-hexane, ethyl acetate, fetal bovine serum (FBS), 0.25% trypsin/EDTA, phosphate buffer saline (PBS), 3-(4,5-dimethylthiazol-2-yl)-2,5-diphenyltetrazolium-bromide (MTT), trypan blue, propidium iodide (PI) and crystal violet were purchased from Sigma-Aldrich (St. Louis, MO, USA). Ethanol of analytical grade with 99.5% purity was procured from R&M Marketing (Essex, England, UK); meanwhile, ethanol of molecular grade, dimethyl sulfoxide (DMSO), propidium iodide (PI), 4,6-diamidino-2-phenylindole-dihydrochloride (DAPI) and RNase A were purchased from Thermo Fisher Scientific (Waltham, MA, USA). β-Mercaptoethanol was purchased from Calbiochem (San Diego, CA, USA), whereas cisplatin, eleutherine, and isoeleutherine were obtained from BioCrick (Chengdu, China). Reagents like penicillin-streptomycin and amphotericin B were obtained from Corning (Union City, CA, USA), whereas Roswell Park Memorial Institute (RPMI-1640) was purchased from Nacalai Tesque (Nakagyo-ku, Kyoto, Japan). ReverTra Ace™ qPCR RT Master Mix with gDNA Remover kit and THUNDERBIRD™ SYBR^®^ qPCR Mix were purchased from Toyobo (Kita Ward, Osaka, Japan) whereas, FavorPrep™ Blood/Cultured Cell RNA Mini Kit was obtained from Favorgen (Nong-Ke Rd, Ping-Tung, Taiwan). CellTiter-Glo^®^ 3D reagent was purchased from Promega (Madison, WI, USA), nCounter PanCancer Pathway Panel kit was purchased from NanoString (Seattle, WA, USA), Hoechst 33342 was purchased from Biotium (Fremont, CA, USA), and PE/Cy7-conjugated CD44 mouse monoclonal antibody was purchased from Abcam (Cambridge, England, UK).

### 3.2. Raw Material and Sample Preparation

*Eleutherine bulbosa* bulbs were purchased from Segamat, Johor, Malaysia, and identified by the Biodiversity Unit, Institute of Bioscience, UPM Serdang, Selangor, Malaysia (Voucher specimen: MFI 0205/21). The bulbs were thoroughly washed under running tap water, sliced, and dried overnight in a 40 °C warm air oven. Next, the dried sliced bulbs were grounded into a fine powder, sieved, and stored at 4 °C for further use.

### 3.3. Ethanol Extraction and Liquid-Liquid Fractionation

Ethanol extraction of *E. bulbosa* bulbs was performed, as reported by Kamarudin et al. (2020) [49]. Following its preparation, finely powdered *E. bulbosa* (10 g) were mixed with 146 mL of 90% ethanol and then heated for 70 min at 45 °C in a water bath. Next, the ethanol extract was filtered, evaporated in a rotary evaporator, and dried overnight in a 40 °C oven.

**Figure 12:**
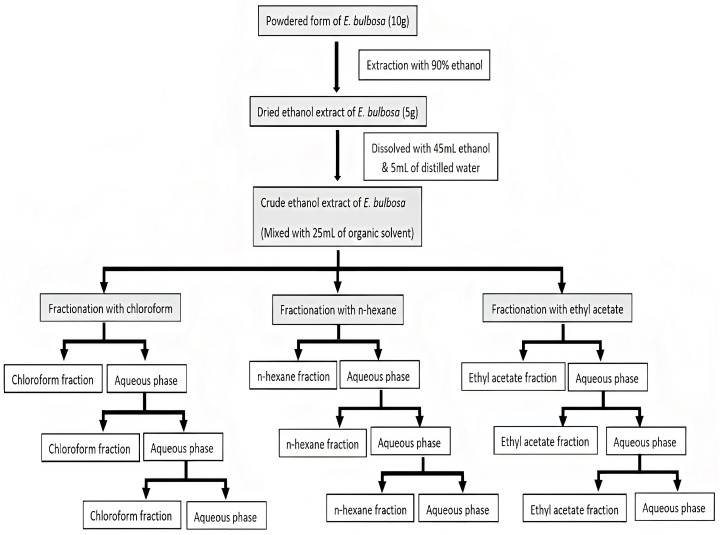
Liquid-liquid fractionation of crude ethanol extract of *E. bulbosa*.

The ethanol extract was then subjected to a liquid–liquid fractionation, as described by Lestari et al. (2019) and Park and Kim (2017), with minor changes [47,75]. Dried ethanol extract (5 g) was dissolved in a mixture of ethanol (45 mL) and distilled water (5 mL) to produce the crude ethanol extract. The mixture was fractionated using three organic solvents to obtain three fractions: chloroform, n-hexane and ethyl acetate. Initially, 25 mL of chloroform solvent was added to the crude ethanol extract and placed into a separating funnel to separate the chloroform and water fraction. The water fraction was mixed with another 25 mL of chloroform solvent and the fractionation process was repeated three times. Next, the chloroform fraction was evaporated using a rotary evaporator and dried overnight in a 40 °C oven. Moreover, the fresh crude ethanol extract was then fractionated with n-hexane and ethyl acetate separately to create the n-hexane and ethyl acetate fractions. The details of the liquid–-liquid fractionation protocol are presented in Figure 12.

### 3.4. 2D Culture Condition

#### 3.4.1. Cell Lines and Culture

Human lung adenocarcinoma A549 cells (ATCC^®^ CCL-185™) and human lung fibroblast MRC-5 cells (ATCC^®^ CCL-171™) were used in this study and purchased from the American Type Culture Collection (ATCC). Both cell lines were grown as monolayers in RPMI-1640 media, supplemented with 10% fetal bovine serum (FBS), 1% penicillin-streptomycin, and 0.5% of amphotericin B at 37 °C in an incubator with 5% CO_2_.

#### 3.4.2. Cytotoxic Activity Assay

The MTT assay was conducted on the monolayer of A549 and MRC-5 cells to assess the cytotoxic effect of *E. bulbosa* fractions after 72 h of treatment, as published by Kamarudin et al. (2022), with a few changes [19]. This study selected a 72-h timeframe because prolonged treatment exposure can cause cumulative toxic effects on the cells. Initially, 4 × 10^3^ cells in 100 μL were seeded into each well of a 96-well plate for optimum growth and incubated overnight in a 37 °C incubator. The A549 cells were treated with chloroform fraction, n-hexane fraction, and ethyl acetate fraction with a series of concentrations (500, 250, 125, 62.5, 31.25, 15.63, 7.8 μg/mL) for 72 h. Furthermore, cisplatin was used as a positive control in this study. A549 cells were treated with cisplatin at 50, 25, 12.5, 6.25, 3.125, 1.56, and 0.78 μg/mL concentrations. On the other hand, the chloroform fraction, which is the most cytotoxic fractionated of *E. bulbosa* against A549, was also tested for cytotoxicity against MRC-5 cells through a series of concentrations (500, 250, 125, 62.5, 31.25, 15.63, 7.8 μg/mL) for 72 h. At the end of the treatment, 20 μL of 5 mg/mL MTT solution was added and incubated for 3 h in the dark at 37 °C in an incubator. Next, 100 μL of DMSO was added to each well to solubilize the formazan purple crystal. The absorbance values were measured at 570 nm using an ELISA microplate reader (Synergy™ H1 Multi-Mode, BioTek, Winooski, VT, USA), and all the measurements were conducted in triplicate. The percentage of cytotoxicity is represented by the percentage of cell viability with the formula as follows:

$$\%\;\mathrm{cell}\;\mathrm{viability}=\frac{Absorbance\;value\;of\;sample}{Absorbance\;value\;of\;control}\times 100$$

The cytotoxic activity was determined as the mean concentration of sample needed to inhibit 50% of the cell growth (IC_50_). Following determination of IC_50_ values of the fractions in A549 and MRC-5 cells, the selectivity index (SI) of the fractions was calculated in accordance as follows:

$$\mathrm{Selectivity}\;\mathrm{index}\;(\mathrm{SI})=\frac{IC50\;of\;normal\;cells}{IC50\;of\;cancer\;cells}\times 100$$

#### 3.4.3. Clonogenic Survival Assay

The clonogenic assay studies cell survival based on the ability of a single cell to form a colony. This assay followed the same techniques as reported by Moritz et al. (2014) with modifications [76]. First, 4 × 10^3^ A549 cells were seeded into each well of a 6-well plate and grown overnight. Next, the cells were treated for 72 h with chloroform fraction at 30.01 μg/mL, which is the most cytotoxic against A549 or cisplatin at 2.61 μg/mL. At the end of treatment, the cells were washed with PBS, fresh complete growth media were added, and the cells were further incubated for ten days in the incubator. Then, the cells were washed with PBS, fixed with 70% ethanol for 15 min and stained with 0.5% (*w*/*v*) crystal violet for 20 min. The plates were washed with running tap water and dried at room temperature before counting stained colonies using an inverted phase contrast microscope (ZEISS Axio Vert.A1, ZEISS, Oberkochen, Baden-Wurttemberg, Germany). In the present study, the untreated cells served as the positive control. 

#### 3.4.4. Cell Death Analysis

This analysis utilized the double staining of Hoechst 33342 and propidium iodide (PI) solution on A549 cells as described by Yong et al. (2015) with minor modifications [77]. The A549 cells were seeded at a density of 1.2 × 10^6^ cells/well in a 6-well plate for optimum growth and incubated overnight at 37 °C in a CO_2_ incubator. The cells were then treated for 72 h with cisplatin at 2.61 μg/mL or chloroform fraction at 30.01 μg/mL. After treatment, the cells were washed twice with ice-cold PBS. Then, 1 mL of ice-cold PBS with 10 μL of Hoechst 33342 and 5 μL of PI were added to the cells and incubated in the dark for 15 min at 37 °C. The stained cells were then washed with PBS and observed under a fluorescence microscope (Olympus CKX41 Zeiss Axio Vert.A1) equipped with ZEN Lite software (Zeiss, Oberkochen, Baden-Wurttemberg, Germany). 

### 3.5. GC-MS Analysis

In this analysis, 10 mg of chloroform fraction was dissolved in 1 mL of DMSO and passed through a 0.45 μm nylon syringe filter to create a stock solution of 10 mg/mL. Next, 1 μL of the chloroform fraction (10 mg/mL) was analyzed using GC (Shimadzu Gas Chromatography system GC-2010 Plus, Nakagyo-ku, Kyoto, Japan) and MS (GC–MS-QP 2010 Ultra) as described by Rani and Bindu (2016) with slight modifications [78]. The GC-MS instrument used a Rxi™-5ms fused silica capillary low polarity column (30.0 m in length, 0.25 mm in diameter, and 0.25 μm in thickness), and helium served as the carrier gas at a flow rate of 0.80 mL/min. The injection temperature was set at 250 °C, and the oven temperature was programmed as follows: an initial temperature of 50 °C which increased to 300 °C at a rate of 3 °C/min after a 10-min hold. The mass spectrometer achieved a spectral range of 40–700 *m*/*z* using electron ionization (EI) mode and an ion source temperature of 200 °C. 

The chemical components of the chloroform fraction were determined and verified by comparing their mass spectral fragmentation patterns to the retention time similarity indices in the Wiley229, FFNSC1.3, NIST11, and NIST14 libraries with cross-references to the reported literature. The relative area of the peaks in the chromatogram represents the abundance of each component in the fraction. Any GC-MS chromatographic peak of an unidentified compound was analyzed and compared with the standard compounds, particularly eleutherine and isoeleutherine, as described earlier to confirm the identification of compounds.

### 3.6. 3D Culture Condition

#### 3.6.1. Generation of Lung Cancer Spheroids

The A549 cells were seeded at a density 4 × 10^3^ cells/well in the 96-well round bottom ultra-low attachment plates (Corning, Union City, CA, USA) at 200 μL per well of complete growth media and incubated for 72 h at 37 °C in a CO_2_ incubator without disturbing the plates to generate the spheroid. The cells self-aggregated and formed spheroids.

#### 3.6.2. Cytotoxicity Activity on Lung Cancer Spheroids

The CellTiter-Glo^®^ 3D reagent was used following the manufacturer’s instructions. A549 spheroids were treated with the chloroform fraction through a series of concentrations (500, 250, 125, 62.5, 31.25, 15.63, 7.8 μg/mL) for 72 h in a CO_2_ incubator. Next, 100 μL of CellTiter-Glo^®^ 3D solution was added to each well. The plates were shaken for 5 min using a microplate reader to lyse the spheroids and incubated at room temperature for 25 min to stabilize the luminescent signal, protected from the light. The lysed spheroids were transferred from the 96-well round bottom ultra-low attachment plate to the 96-well white-opaque flat bottom plate. The resultant luminescence signals were recorded using a microplate reader (Synergy™ H1 Multi-Mode, BioTek, Winooski, Vermont, USA). The percentage of cell viability represents the percentage of cytotoxicity. The cytotoxic activity was expressed as the mean concentration of the sample required to inhibit 50% of cell growth (IC_50_).

#### 3.6.3. Microscopy Analysis of Lung Cancer Spheroids

##### Spheroid Size Analysis

A549 spheroids were treated with the chloroform fraction for 72 h at 78.92 μg/mL. After the treatment, the untreated and treated spheroids were examined, and the photographs were captured using bright-field imaging on a fluorescence microscope with ZEN Lite software. The spheroid size was analysed using Image J software (NIH, Bethesda, MD, USA).

##### Cell Death Analysis of Spheroid Using Hoechst 33342/PI Staining

The chloroform fraction was exposed to A549 spheroids for 72 h at 78.92 μg/mL. Next, the spheroids were collected and washed using PBS by centrifuging the spheroids at a speed of 500 rpm for 3 min. The spheroids were fixed for 30 min at 4 °C with 70% ice-cold ethanol. Then, the spheroids were washed and stained with 10 µL of Hoechst 33342 and 5 μL of PI solutions in the dark for 15 min at 37 °C. Each spheroid was transferred into each well of a 24-well plate containing 1 mL of PBS after the stained spheroids were rewashed. The spheroids were observed, and the fluorescence images were captured using the fluorescence microscope equipped with ZEN Lite software.

#### 3.6.4. Flow Cytometry Analysis on Lung Cancer Spheroids

##### Immunophenotyping of CD44 Lung Cancer Stem Cells

The A549 spheroids were treated with the chloroform fraction at 78.92 μg/mL for 72 h. Next, the cells were extracted from spheroids to quantify the CD44+ cell population. According to the manufacturer’s recommendation, 100 μL of FACS buffer and 1 μL of PE/Cy7-conjugated CD44 mouse monoclonal antibody solution was added to the cells and incubated for 30 min on ice in the dark. The cells were rewashed and resuspended in 1 mL of FACS buffer and passed through a 40 μm cell strainer into FACS tubes to eliminate the remaining cell clusters. The cells were placed on ice to preserve viability before being analyzed using a BD LSRFortessa^®^ flow cytometer.

##### Cell Cycle Analysis

Cells were extracted from A549 spheroids after 72 h of treatment with the chloroform fraction at 78.92 μg/mL to quantify the cell cycle. According to the manufacturer’s recommendations, 500 μL of PBS with 5 μL of PI and 2 μL of RNase A were added and incubated in the dark at room temperature for 30 min. After incubation, the cells were transferred into the FACS tube before being analysed. The outcomes of the cell population analysis in each stage of the cell cycle were performed in triplicate using a BD LSRFortessa^®^ flow cytometer.

#### 3.6.5. Quantitative Real-Time Polymerase Chain Reaction (qPCR)

Total RNA of untreated and treated A549 spheroids was extracted using the FavorPrep™ Blood/Cultured Cell RNA Mini Kit according to the manufacturer’s instructions after 72 h of treatment with the chloroform fraction at 78.92 μg/mL. A NanoDrop spectrophotometer was used to assess RNA quality and quantity. The cDNA was synthesised from the RNA using ReverTra Ace™ qPCR RT Master Mix with gDNA Remover kit following the manufacturer’s instructions. The resulting cDNA strand was quantitatively determined by the THUNDERBIRD™ SYBR^®^ qPCR Mix, following the manufacturer’s instructions. Bio-Rad CFX-96TM Real-Time System (Bio-Rad, Hercules, CA, USA) and its software were utilized to run a cycling program following the manufacturer’s instructions: 1 cycle of polymerase activation and DNA denaturation at 95 °C for 1 min, followed by 39 cycles of denaturation for 15 s at 95 °C, annealing at 60 °C for 15 s, and the extension step at 72 °C for 45 s. The melting curve analysis was set at 65 °C to 95 °C with an increment of 0.5 °C for 5 s. The primer sets utilized in this experiment are listed in Table 4. The Livak method (2^−ΔΔCT^) was used to determine the relative mRNA expression in triplicate, with glyceraldehyde 3-phosphate dehydrogenase (GAPDH) as the internal control.

### 3.7. Statistical Analysis

The statistical analyses were performed using GraphPad Prism version 9. Each experiment was conducted in triplicate, and the collected data were presented as mean ± standard deviation (SD). The unpaired Student’s *t*-test (two-tailed) was used to analyse the differences between the means, and *p* < 0.05 represents a statistically significant difference. The one-way analysis of variance (ANOVA) was utilized to evaluate if there was a statistically significant difference between groups at the 95% confidence level (*p* < 0.05).

## 4. Conclusions

The chloroform fraction of *E. bulbosa* was the most cytotoxic against lung cancer cells (A549) and exhibited selective toxicity toward lung cancer cells since it was weakly cytotoxic to the normal lung cells (MRC-5). This finding showed that the chloroform fraction of *E. bulbosa* has the therapeutic potential in modulating anticancer activity against lung cancer cells while minimizing cytotoxic effects against normal cells. Moreover, the chloroform fraction of *E. bulbosa* exhibits its antiproliferative effect by inhibiting the capacity of A549 cells to survive by reducing their clonogenic capability and thus inducing apoptosis of these A549 cells. The chloroform fraction also inhibited the growth of A549 spheroids by suppressing the spheroid size, inducing apoptosis, reducing the proportion of CD44 lung cancer stem cells, causing arrest at the S phase of the cell cycle, and suppressing the expression of the pluripotency genes (SOX2 and MYC) that control the stemness of lung cancer stem cells. In addition, further analysis by GC-MS revealed the presence of several active compounds that may contribute to the anticancer effects of the chloroform fraction, including the major compounds of eleutherine and isoeleutherine. However, the presence of these compounds needs to be confirmed using extensive identification analyses, and the predicted anticancer effects of these compounds also need to be validated. Nevertheless, based on the findings of this study, the chloroform fraction of *E. bulbosa* may be useful as a chemopreventive agent in treating lung cancer.

## Figures and Tables

**Table 1:** IC_50_ values of three fractions of *E. bulbosa* and cisplatin. All fractions and cisplatin were tested against A549 cells, whereas only the chloroform fraction was tested against MRC-5 cells.

Fraction	IC_50_ (μg/mL)		Selectivity Index (SI)
	A549 Cells	MRC-5 Cells	
Chloroform	30.01 ± 2.14	102.2 ± 1.78	3.4
n-Hexane	126.60 ± 4.15	-	-
Ethyl acetate	83.44 ± 2.14	-	-
Cisplatin	2.61 ± 0.09	-	-

The significant difference between chloroform, n-hexane, and ethyl acetate fractions was compared using an ANOVA. The level of significance is indicated as *p* < 0.0001. The mean difference between the chloroform fraction treated on A549 and MRC5 was determined using the Student’s *t*-test. The level of significance is indicated as *p* < 0.001. The data are represented as IC_50_ values (mean ± SD) from three independent experiments (*n* = 3).

**Table 2:** List of identified compounds in the chloroform fraction of *E. bulbosa* determined by GC-MS analysis. The abundance of each compound detected in the chloroform fraction of *E. bulbosa* is indicated by the peak area in the chromatogram.

No	Retention Time (min)	Compound Name	Molecular Formula	Molecular Weight (MW)	Peak Area (%)	Similarity Index (%)
1	4.1663	2,3-butanediol	C_4_H_10_O_2_	90	0.38	97
2	4.3425	2,3-butanediol, [R-R *, R *]	C_4_H_10_O_2_	90	0.47	97
3	50.8417	n-hexadecanoic acid	C_16_H_32_O_2_	256	1.49	94
4	51.9708	Hexadecanoic acid, ethyl ester	C_18_H_36_O_2_	284	0.42	78
5	55.4617	Unknown	N/A *	N/A	2.36	N/A
6	56.3982	9,12-octadecadienoic acid	C_18_H_32_O_2_	280	0.56	83
7	56.8070	Benzeneethanal, 4-[1,1-dimethylethyl]	C_12_H_16_O	176	0.78	83
8	57.3080	14-methyl-8-hexadecyn-1-ol	C_17_H_32_O	252	1.02	89
9	59.5095	Unknown	N/A	N/A	36.69	N/A
10	60.8740	Unknown	N/A	N/A	17.25	N/A
11	64.8472	Propanedinitrile, [(3,4,5-trimethoxyphenyl) methylene]	C_13_H_12_N_2_O_3_	244	20.92	78
12	67.1923	Hexadecanoic acid, 2-hydroxy-1-(hydroxyethyl) ethyl ester	C_19_H_38_O_4_	330	0.65	83
13	67.8215	Unknown	N/A	N/A	1.02	N/A
14	68.7435	Unknown	N/A	N/A	2.91	N/A
15	69.2533	Unknown	N/A	N/A	2.42	N/A
16	71.8457	Ethyl linoleate	C_20_H_36_O_2_	308	0.97	81
17	73.1697	Unknown	N/A	N/A	7.49	N/A
18	75.8435	Unknown	N/A	N/A	0.42	N/A
19	85.3685	Stigma sterol	C_29_H_48_O	412	0.83	80
20	86.8377	Stigmast-5-en-3-ol, (3.beta.,24s)-(CAS) clionasterol	C_29_H_50_O	414	0.95	84

* N/A is known as non-available.

**Table 3:** The average size of untreated and chloroform fraction-treated spheroids after 72 h of treatment.

Cell Line	Average Size of Spheroid (μm) (Mean ± SD)	
	Untreated	Treated
A549	492.36 ± 10.62	422.58 ± 9.43

The mean difference between the two groups was determined using the Student’s *t*-test. The level of significance is indicated as *p* < 0.01. The data were represented as mean ± SD from three independent experiments (*n* = 3).

**Table 4:** The nucleotide sequences of primers used in qPCR.

Genes	Accession Number	5′-3′ Sequence	
GAPDH (control)	NM_002046.7	Forward Reverse	GTCATCCCTGAGCTGAACGG CCACCTGGTGCTCAGTGTAG
MYC	NM_002467.6	Forward Reverse	CATCAGCACAACTACGCAGC GCTGGTGCATTTTCGGTTGT
SOX2	NM_003106.4	Forward Reverse	GCCCTGCAGTACAACTCCAT GACTTGACCACCGAACCCAT

## Data Availability

Data are contained within the article.
